# Comparative Microarray Analysis of Intestinal Lymphocytes following *Eimeria acervulina*, *E. maxima*, or *E. tenella* Infection in the Chicken

**DOI:** 10.1371/journal.pone.0027712

**Published:** 2011-11-28

**Authors:** Duk Kyung Kim, Hyun Lillehoj, Wongi Min, Chul Hong Kim, Myeong Seon Park, Yeong Ho Hong, Erik P. Lillehoj

**Affiliations:** 1 Animal Parasitic Diseases Laboratory, Animal and Natural Resources Institute, Beltsville Agricultural Research Center, United States Department of Agriculture, Agricultural Research Service, Beltsville, Maryland, United States of America; 2 College of Veterinary Medicine and Research Institute of Life Science, Gyeongsang National University, Jinju, Korea; 3 Department of Animal Science and Technology, Chung-Ang University, Anseong, Korea; 4 Department of Pediatrics, University of Maryland School of Medicine, Baltimore, Maryland, United States of America; Auburn University, United States of America

## Abstract

Relative expression levels of immune- and non-immune-related mRNAs in chicken intestinal intraepithelial lymphocytes experimentally infected with *Eimeria acervulina*, *E. maxima*, or *E. tenella* were measured using a 10K cDNA microarray. Based on a cutoff of >2.0-fold differential expression compared with uninfected controls, relatively equal numbers of transcripts were altered by the three *Eimeria* infections at 1, 2, and 3 days post-primary infection. By contrast, *E. tenella* elicited the greatest number of altered transcripts at 4, 5, and 6 days post-primary infection, and at all time points following secondary infection. When analyzed on the basis of up- or down-regulated transcript levels over the entire 6 day infection periods, approximately equal numbers of up-regulated transcripts were detected following *E. tenella* primary (1,469) and secondary (1,459) infections, with a greater number of down-regulated mRNAs following secondary (1,063) vs. primary (890) infection. On the contrary, relatively few mRNA were modulated following primary infection with *E. acervulina* (35 up, 160 down) or *E. maxima* (65 up, 148 down) compared with secondary infection (*E. acervulina*, 1,142 up, 1,289 down; *E. maxima*, 368 up, 1,349 down). With all three coccidia, biological pathway analysis identified the altered transcripts as belonging to the categories of “Disease and Disorder” and “Physiological System Development and Function”. Sixteen intracellular signaling pathways were identified from the differentially expressed transcripts following *Eimeria* infection, with the greatest significance observed following *E. acervulina* infection. Taken together, this new information will expand our understanding of host-pathogen interactions in avian coccidiosis and contribute to the development of novel disease control strategies.

## Introduction

Avian coccidiosis is caused by seven species of *Eimeria* protozoa (*E. acervulina*, *E. maxima*, *E. tenella*, *E. mitis*, *E. necatrix*, *E. praecox*, and *E. brunetti*) that differ in pathogenicity and immunogenicity [1], [2]. The life cycles of all *Eimeria* species are of the monoxenous sporozoan type. Generally, infection develops following ingestion of sporulated oocysts and release of sporozoites, which subsequently invade intestinal epithelial cells. Through asexual reproduction, gametes are formed and fertilized to produce a zygote, which matures into an oocyst, ruptures the host cell, and is excreted in the feces. The entire cycle normally develops over the course of 4–6 days, depending on the species [3].

*Eimeria* infection inflicts significant economic losses to the commercial poultry industry due to decreased nutrient absorption, retarded growth rate, reduced egg production, and mortality [4], [5]. Although prophylactic chemotherapy has been traditionally used for disease control, the emergence of drug-resistant parasites and legislative bans on the use of in-feed antibiotic growth promoters and non-therapeutic antimicrobial feed additives encourages the development of alternative coccidiosis control strategies [5]. Accordingly, there has been great interest in understanding the host-pathogen interactions at the cellular and molecular levels and to identify effector molecules mediating protective immunity to *Eimeria*.

Functional genomics and bioinformatics technologies have recently emerged as powerful technologies for investigation of host-pathogen interactions during avian coccidiosis [6], [7], [8], [9], [10], [11]. New candidate genes which influence host immune responses to *Eimeria* have been identified using chicken macrophage and lymphocyte cDNA microarrays [6], [7], [10], [11], [12], [13], [14]. Host genetic alterations following *Eimeria* infection have been investigated employing a variety of new microarray data mining tools [15], [16], [17], [18], [19]. However, a comparative analysis of gene expression in gut lymphocytes in response to infection by different species of *Eimeria* has not been reported, which would aid in the understanding of species-related differences in parasite pathogenicity and immunogenicity. Therefore, this study was conducted to compare the global gene transcripts of the three species of coccidia that most commonly infect commercial poultry, *E. acervulina*, *E. maxima*, and *E. tenella*.

## Materials and Methods

### Experimental animals and *Eimeria* infection

All experiments were approved by the Beltsville Agriculture Research Center Small Animal Care and Use Committee (Protocol #09-019). One week-old chickens were uninfected (negative control) or were orally inoculated with sporulated oocysts of *E. acervulina*, *E. maxima*, or *E. tenella* (1.0×10^4^oocysts/bird). One week later, the infected chickens were challenged with an identical inoculum of the homologous parasite. Intestinal samples were collected daily from 5 birds in a treatment group at from 1 to 6 days post-infection (DPI) following primary and secondary infections. Cecum, duodenum, and jejunum were collected from the birds challenged with *E. acervulina*, *E. maxima*, and *E. tenella*, respectively.

### RNA extraction and aminoallyl-labeled RNA preparation

Intestines were cut longitudinally and washed three times with ice-cold Hanks' balanced salt solution (HBSS) containing 100 U/ml of penicillin and 100 mg/ml of streptomycin (Sigma, St. Louis, MO). The mucosal layer of intestine was carefully scraped using a cell scraper and intraepithelial lymphocytes (IELs) were isolated by Percoll density gradient centrifugation as described (Min *et al.*, 2005). Total RNA was isolated using Trizol (Invitrogen, Carlsbad, CA), and RNAs from animals in the same treatment group were pooled and purified using the RNeasy Mini RNA Purification Kit (Qiagen, Valencia, CA). Aminoallyl-labeled RNA was prepared using the Amino Allyl Message Amp II aRNA Amplification Kit (Ambion, Austin, TX) according to the manufacturer's instruction. Two of 20 µg aliquots of each aminoallyl-RNA sample were fluorescently labeled with AlexaFluor 555 or AlexaFluor 647 (Invitrogen). RNA concentrations and labeling efficiencies were determined spectrophotometrically.

### Mircroarray hybridization

The avian IEL array (AVIELA) consists of 10,162 spots representing duplicates of cDNAs from chicken IELs, immune-related cDNAs from lipopolysaccharide-activated HD11 chicken macrophages, and direct PCR clones of selected chicken cytokine and chemokine genes [8], [11], [12]. Uninfected control samples and one of the 3 infection group samples were labeled with different fluorescent dyes and hybridized simultaneously on the same slide using a reference design with a dye swap protocol [20]. Hybridizations were performed overnight at 50°C using HybIt hybridization buffer (TeleChem, Sunnyvale, CA) in ArrayIt reaction cassettes as described [11]. Following hybridization, the slides were rinsed in 0.5×SSC, 0.01% SDS and washed once for 15 min at room temperature in 0.2×SSC, 0.2% SDS at 50°C, 3 times for 1 min at room temperature in 0.2×SSC, and 3 times for 1 min at room temperature in distilled water.

### Microarray scanning and data analysis

Images were acquired by laser confocal scanning using a ScanArray Lite microarray analysis system (Perkin-Elmer, Boston, MA) at a resolution of 10 µm. A 16-bit TIFF image was generated for each channel corresponding to the Alexa Fluor 555 and Alexa Fluor 647 dyes. The scanned microarray images for each channel were overlaid and fluorescent intensities were quantified using ScanArray Express version 3.0 software (Perkin-Elmer). Spots were detected using an adaptive circle algorithm in the ScanArray program and all spots were visually confirmed. The MIDAS 2.19 of the TM4 package (http://www.tigr.org) was used to qualify and normalize the array data. The poor-quality channel tolerance policy was stringent and the signal-noise threshold was 2.0. Two-step normalization, total intensity, and global LOWESS (locally weighted regression and smoothing scatter) plot methods were applied followed by standard deviation (SD) regularization between blocks and slides. GeneSpring GX 11.0 (Silicon Genetics, Redwood, CA) was used to perform statistical analyses of the qualified and normalized array data. Flag information was applied to filter bad spots with genes missing more than 50% of their values because of low signal-to-noise ratio. Student's t-test and ANOVA analysis by parametric test with multiple testing correction (Benjamini and Hochberg False Discovery Rate) were applied for data normalization to control values and to compare values between different infection groups. All microarray data in this study adheres to the reporting guidelines provided by MIAME and have been submitted online into the Gene Expression Omnibus (GEO) (Series #GSE31213; Samples #GSM773800-GSM773871).

### Quantitative RT-PCR

Gene expression changes observed by microarray analysis were confirmed by quantitative (q)RT-PCR as described [7]. Equivalent amounts of the same RNA samples used for microarray hybridizations from 1–6 days post-primary or post-secondary infections with the different *Eimeria* species were pooled. The RNAs were reverse transcribed using the StrataScript First Strand Synthesis System (Stratagene, La Jolla, CA). Amplification and detection were carried out using the Mx3000P system and Brilliant SYBR Green qRT-PCR master mix (SABioscience, Frederick, MD). Oligonucleotide primers are listed in Table 1. Standard curves were generated using log_10_ diluted standard RNA and the levels of individual transcripts were normalized to those of GAPDH by the Q-gene program [21]. The fold changes were calculated in the normalized mRNA levels between the uninfected control group and each infection groups. Each analysis was performed in triplicate and the comparisons of the mean values were performed by one-way analysis of variance (ANOVA) followed by the Duncan's multiple range test using SPSS software (SPSS 15.0 for Windows, Chicago, IL).

**Table 1:** Primers used for quantitative RT-PCR.

Symbol	Gene Name	Forward Primer (5′→3′)	Reverse Primer (5′→3′)	GenBank Accession No.
ADA	Adenosine deaminase	CATTCGGCCAGAAACAATCT	GTAGACGACGCCTTCCTTTG	NM_001006290.1
BECN1	Beclin 1, autophagy related	CCAGATGCGTTATGCTCAGA	TTGCCATACGGTACAAGACG	NM_001006332.1
CCL20	Chemokine (C-C motif) ligand 20	GGCTTGAGCACCAAGAGTTT	GGATTTACGCAGGCTTTCAG	NM_204438.1
CD8A	CD8α molecule	AATGGTGTCTCCTGGATTCG	CAGCATCTGGTTGATGTTGG	NM_205235.1
TLR4	Toll-like receptor 4	CCTGAAATGGGTCAAGGAAA	TTACACCCACTGAGCAGCAC	NM_001030693.1
IL-6	Interleukin-6	CAAGGTGACGGAGGAGGAC	TGGCGAGGAGGGATTTCT	AJ309540
GAPDH	Glyceraldehyde-3-phosphate dehydrogenase	GGTGGTGCTAAGCGTGTTAT	ACCTCTGTCATCTCTCCACA	K01458

### Bioinformatics analysis

IEL cDNA sequences in the AVIELA were mapped to the chicken genome reference assembly (version 2.1) and to reference RNA and protein sequences using National Center for Bioinformatics Institute (NCBI) Blast (version 2.2.13). The networks and pathway information of genes which were differentially expressed were analyzed by Ingenuity Pathways Analysis (IPA) software (Ingenuity Systems, http://www.ingenuity.com). The dataset containing gene identifiers mapped to UniGene IDs in the chicken (*Gallus gallus*) database (Build #43) and corresponding expression values were uploaded into the application. Each identifier was mapped to its corresponding gene object in the Ingenuity knowledge base. Both up- and down-regulated identifiers were defined as value parameters for the analysis. These genes, called focus genes, were overlaid onto a global molecular network developed from information contained within IPA. Functional gene analysis was performed to identify the biological functions and canonical pathways of genes from the mapped datasets. The Fischer's exact test was used to calculate P values to assess the probability that each biological function and pathway assigned to that dataset. Biological functions and pathways of those focus genes were generated based on their connectivity.

## Results

### Comparison of mRNA expression levels following infection with *E. acervulina*, *E. maxima*, or *E. tenella*

**Figure 1:**
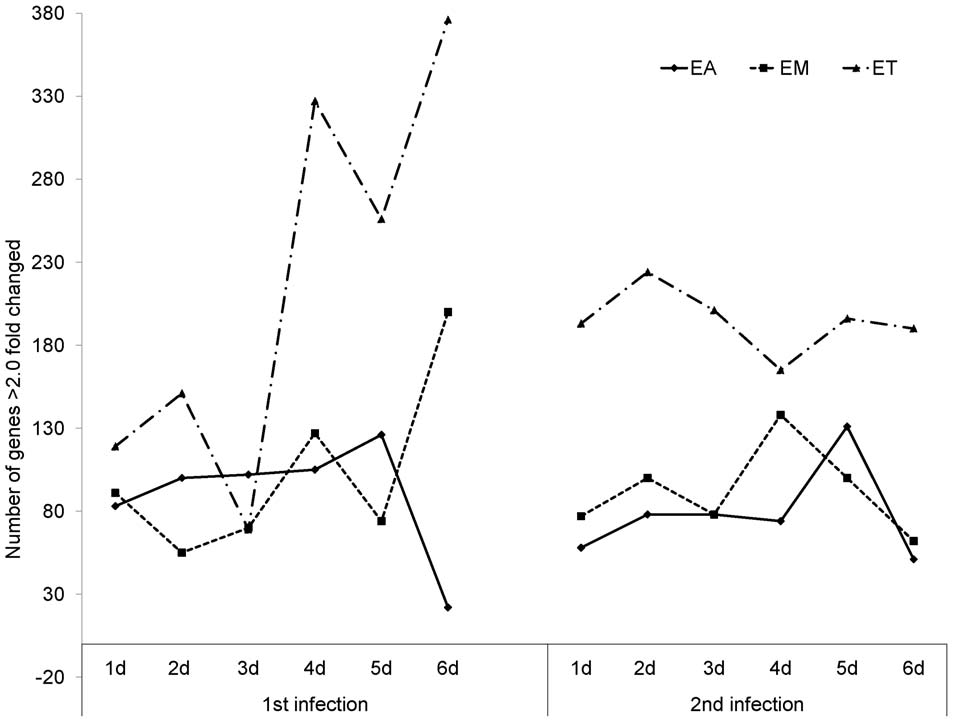
Comparison of the number of intestinal lymphocyte transcripts with <2.0-fold altered levels following primary (1^st^) and secondary (2^nd^) infections by *E. acervulina* (EA), *E. maxima* (EM), or *E. tenella* (ET) compared with uninfected controls (P<0.05).

**Figure 2:**
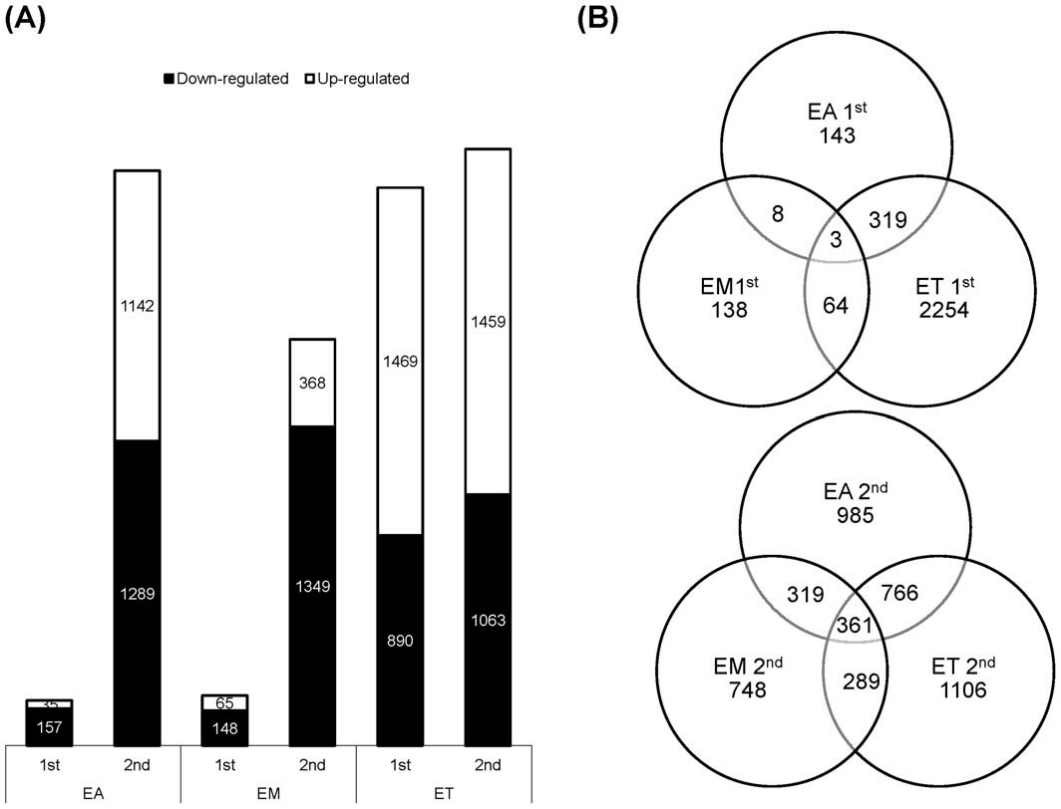
The effects of primary (1^st^) and secondary (2^nd^) infections by *E. acervulina* (EA), *E. maxima* (EM), or *E. tenella* (ET) compared with uninfected controls (P<0.0005) on the intestinal lymphocyte transcripts. (A) The number of transcripts differentially expressed and (B) the number of the overlapping transcripts differentially expressed following 1^st^ and 2^nd^ infections by EA, EM, or ET.

**Figure 3:**
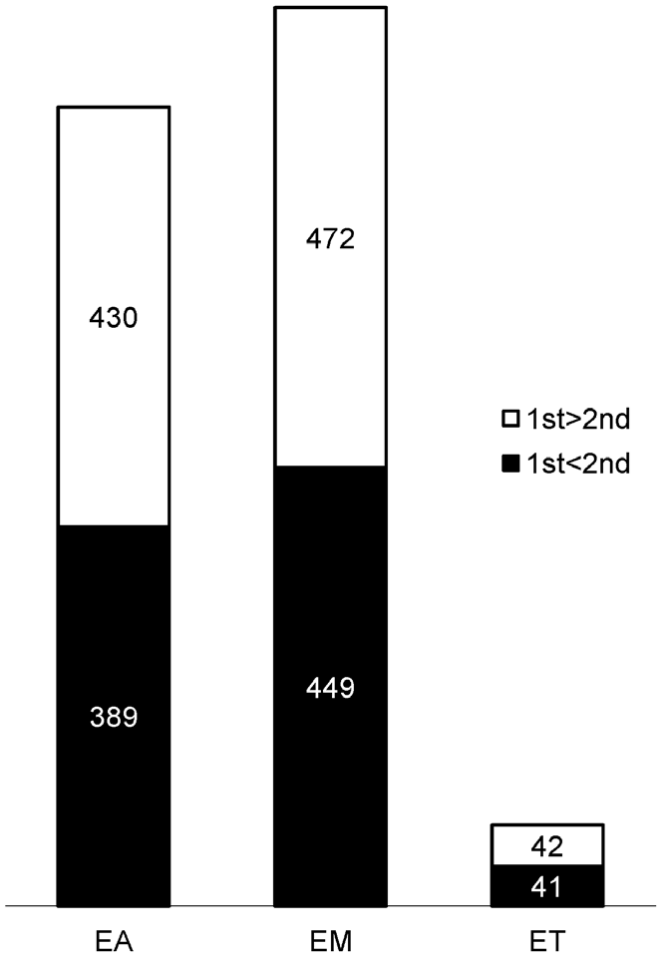
Comparison of the number of intestinal lymphocyte transcripts differently expressed when comparing primary (1^st^) and secondary (2^nd^) infections by *E. acervulina*, *E. maxima*, or *E. tenella* (P<0.0005).

**Figure 4:**
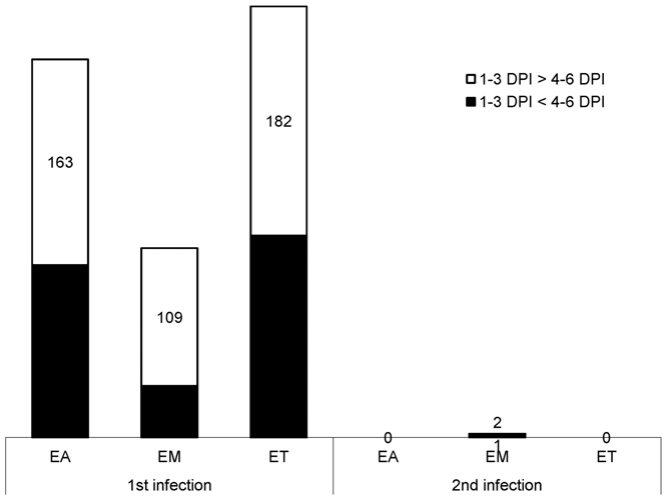
Comparison of the number of intestinal lymphocyte transcripts differentially expressed at 1–3 DPI vs. 4–6 DPI following primary (1^st^) and secondary (2^nd^) infections by *E. acervulina* (EA), *E. maxima* (EM), or *E. tenella* (ET).

Using a cutoff of >2.0-fold differential expression compared with uninfected controls, relatively equal numbers of transcripts were altered at 1, 2, and 3 days post-primary infection with *E. acervulina*, *E. maxima*, or *E. tenella* (Figure 1). By contrast, *E. tenella* elicited the greatest number of altered transcripts at 4, 5, and 6 days post-primary infection, and at all time points following secondary infection. The numbers of transcripts that were significantly (P<0.0005) up- or down-regulated over the entire 6 day infection periods when comparing each infection group with uninfected controls are illustrated in Figure 2A. Approximately equal numbers of up-regulated transcripts were detected following *E. tenella* primary (1,469) and secondary (1,459) infections, with fewer down-regulated mRNAs following primary (890) vs. secondary (1,063) infection. On the other hand, only a small subset of mRNAs were modulated following primary infection with *E. acervulina* (35 up, 157 down) or *E. maxima* (65 up, 148 down) compared with secondary infection (*E. acervulina*, 1,142 up, 1,289 down; *E. maxima*, 368 up, 1,349 down). Figure 2B showed the number of the differentially expressed genes that were overlapped between the infections of three different *Eimeria* species. In the primary and secondary infections, the genes commonly changed by three species were three and three hundred sixty one, respectively. When comparing the total number of transcripts altered between 1–6 DPI following primary or secondary infection, the levels of 430 mRNAs were greater in primary vs. secondary infection with *E. acervulina*, and 389 transcript levels were greater in secondary vs. primary infection (Figure 3). For *E. maxima*, 472 transcripts were greater following primary and 449 transcripts were greater after secondary infection. Relatively equal numbers were altered following *E. tenella* infection (42 vs. 41). Next, we compared the numbers of transcripts between early (1–3 DPI) and late (4–6 DPI) primary or secondary infections whose levels were altered compared with uninfected controls (P<0.01). As shown in Figure 4, following primary infection with *E. acervulina*, *E. maxima*, or *E. tenella*, more transcripts were altered between 1–3 DPI compared with 4–6 DPI. By contrast, there were essentially no differences between these two time frames after secondary infection with any of the coccidia parasites. All of the annotated genes that were differentially expressed by three different species of *Eimeria* in primary or secondary infections and between early and late primary infections are shown in Table S1 and Table S2, respectively.

### Quantitative RT-PCR confirmation

The expression patterns observed by microarray hybridization were validated by qRT-PCR with 6 transcripts whose levels were significantly modulated during primary or secondary infection compared with uninfected controls. These genes were adenosine deaminase (ADA), beclin 1, autophagy related (BECN1), chemokine (C-C motif) ligand 20 (CCL20), CD8α (CD8A), Toll-like receptor 4 (TLR4), and interleukin-6 (IL-6). The levels of all 6 transcripts that were up-or down-regulated by microarray analysis also were consistently up- or down-regulated when analyzed by qRT-PCR (Table 2). As previously discussed, the differences in the magnitude of the changes observed might be due to differences in the normalization methods used and/or the different fluorescent dyes used by the two protocols [22].

**Table 2:** Comparison between microarray analysis and quantitative RT-PCR.

Gene Symbol	*Eimeria*	Infection	Microarray^1^	qRT-PCR^1^
ADA	*E. acervulina*	2nd	2.2	2.2
	*E. tenella*	1st	−7.5	−6.7
	*E. tenella*	2nd	−7.6	−10.3
BECN1	*E. acervulina*	2nd	2.3	1.1
	*E. tenella*	1st	−4.5	−1.3
	*E. tenella*	2nd	−4.8	−1.2
CCL20	*E. tenella*	2nd	−5.3	−3.5
CD8A	*E. tenella*	2nd	−3.4	−1.1
TLR4	*E. acervulina*	2nd	−7.6	−1.5
IL6	*E. tenella*	1st	−2.0	−1.2

1 Fold change (P<0.05).

### Biological pathway analysis of differentially regulated transcripts

Biological function analysis using the IPA database was performed for the mRNAs that were differently altered (P<0.0005) following *Eimeria* primary or secondary infections, compared with uninfected controls. In this manner, the transcripts were classified under the categories of “Disease and Disorder” or “Physiological Development and Function”. In the “Disease and Disorder” category, 10 unique biological functions were identified, all of which were recognized following primary infection with one or more of the coccidia parasites (Table 3). A subset of five of these (“Cancer”, “Genetic Disorder”, “Gastrointestinal Disease”, “Infectious Disease”, “Infection Mechanism”) were also identified following secondary infection with all of the denoted *Eimeria* species. In the category of “Physiological Development and Function”, 10 unique biological functions were identified, but only those related to “Cell-mediated Immune Response” and “Hematological System Development and Function” were universally identified following primary *E. acervulina*, *E. maxima*, or *E. tenella* infection (Table 4).

**Table 3:** Biological functions in the category “Disease and Disorder” of the transcripts differentially expressed by *Eimeria* infection.

Infection	*Eimeria*	Biological Function Identified^1^	P value^2^	No. of Genes
1st infection	*E. acervulina*	Cancer	6.75E-05 - 4.94E-02	32
		Gastrointestinal Disease	2.34E-04 - 4.48E-02	15
		Genetic Disorder	2.86E-04 - 3.20E-02	18
		Organismal Injury and Abnormalities	2.23E-03 - 4.48E-02	5
		Connective Tissue Disorders	5.08E-03 - 2.02E-02	4
	*E. maxima*	Connective Tissue Disorders	2.19E-04 - 1.88E-02	4
		Skeletal and Muscular Disorders	2.19E-04 - 3.27E-02	13
		Developmental Disorder	4.57E-04 - 3.89E-02	10
		Reproductive System Disease	4.57E-04 - 3.27E-02	5
		Cancer	7.64E-04 - 4.94E-02	32
	*E. tenella*	Genetic Disorder	6.14E-14 - 6.85E-03	541
		Cancer	1.66E-10 - 6.80E-03	342
		Gastrointestinal Disease	1.17E-06 - 6.80E-03	233
		Infection Mechanism	2.14E-06 - 5.69E-03	130
		Infectious Disease	2.14E-06 - 4.38E-03	138
2nd infection	*E. acervulina*	Cancer	1.44E-10 - 8.10E-03	353
		Genetic Disorder	1.86E-10 - 7.38E-03	537
		Gastrointestinal Disease	1.48E-08 - 7.38E-03	176
		Infectious Disease	1.18E-07 - 6.81E-03	142
		Infection Mechanism	1.25E-06 - 3.48E-03	143
	*E. maxima*	Cancer	2.30E-11 - 1.18E-02	256
		Infectious Disease	3.17E-08 - 1.11E-02	112
		Genetic Disorder	3.63E-08 - 1.18E-02	384
		Infection Mechanism	6.12E-07 - 9.96E-03	115
		Gastrointestinal Disease	2.36E-05 - 1.22E-02	127
	*E. tenella*	Genetic Disorder	9.52E-13 - 1.24E-02	573
		Cancer	1.85E-11 - 1.46E-02	372
		Infectious Disease	7.72E-08 - 1.45E-02	149
		Infection Mechanism	1.10E-07 - 1.40E-02	154
		Gastrointestinal Disease	9.37E-07 - 1.17E-02	255

1 Datasets were analyzed by BioFunction analysis using IPA software. Functions are listed in descending order of statistical significance with the most significant at the top of each *Eimeria* species grouping.

2 P values were calculated using the right-tailed Fisher's exact test.

**Table 4:** Biological functions in the category “Physiological System Development and Function” of the transcripts differentially expressed by primary *Eimeria* infection.

*Eimeria*	Biofucntion^1^	P value^2^	No. of Genes
*E. acervulina*	Hepatic System Development and Function	8.96E-04 - 4.48E-02	3
	Cell-mediated Immune Response	5.08E-03 - 4.96E-02	4
	Endocrine System Development and Function	5.08E-03 - 4.96E-02	4
	Hematological System Development and Function	5.08E-03 - 4.96E-02	7
	Hematopoiesis	5.08E-03 - 4.96E-02	6
*E. maxima*	Tissue Morphology	4.17E-04 - 4.63E-02	9
	Reproductive System Development and Function	7.79E-04 - 4.18E-02	10
	Hematological System Development and Function	9.87E-04 - 4.63E-02	13
	Renal and Urological System Development and Function	1.67E-03 - 2.34E-02	6
	Cell-mediated Immune Response	1.70E-03 - 4.63E-02	8
*E. tenella*	Hematological System Development and Function	2.41E-05 - 6.85E-03	126
	Hematopoiesis	2.41E-05 - 2.82E-03	80
	Cell-mediated Immune Response	4.80E-05 - 2.69E-03	59
	Connective Tissue Development and Function	1.67E-04 - 6.85E-03	54
	Digestive System Development and Function	2.11E-04 - 5.20E-03	18

1 Datasets were analyzed by BioFunction analysis using IPA software. Functions are listed in descending order of statistical significance with the most significant at the top of each *Eimeria* species grouping.

2 P values were calculated using the right-tailed Fisher's exact test.

### Network analysis of differentially regulated transcripts

**Figure 5:**
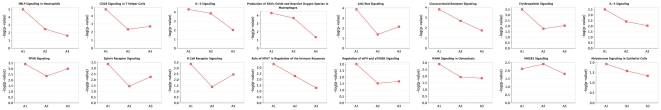
Network analysis of differentially regulated transcripts. The significantly modified signaling pathways (P<0.05) for the transcripts differentially expressed following primary and secondary infections by *E. acervulina* (EA), *E. maxima* (EM), or *E. tenella* (ET). A1; EA, A2; EM, and A3; ET.

Sixteen signal transduction pathways were identified from the differentially expressed transcripts following infection with all three *Eimeria* species, compared with uninfected controls (Figure 5). With the exception of the HMGB1 (high-mobility group protein B1) pathway, the greatest statistical significance was observed between these mRNAs and *E. acervulina* infection for all pathways. Seven of the pathways identified (“CD28 Signaling in T Helper Cells”, “fMLP Signaling in Neutrophils”, “HMGB1 Signaling”, “IL-3 Signaling”, “IL-4 Signaling”, “Production of Nitric Oxide and Reactive Oxygen Species in Macrophages”, “Role of NFAT in Regulation of the Immune Response”) are included in the category of “Cellular Immune Response”, and four pathways are located in the category of “Humoral Immune Response” (“B Cell Receptor Signaling”, “HMGB1 Signaling”, “IL-4 Signaling”, “Role of NFAT in Regulation of the Immune Response”).

Because the comparison of modulated transcripts revealed that the vast majority of alterations between 1–3 DPI and 4–6 DPI were seen following primary, but not secondary, infection (Figure 4), further network analysis was performed using these two time frames. Shown in Table 5 are the associated network functions, and the number of their corresponding focus genes, that were identified by comparison of the altered transcripts at 1–3 DPI with those at 4–6 DPI following primary infection with the three coccidia parasites. Focus genes are those identified and mapped to corresponding gene objects in the IPA database, and whose expression is significantly differentially regulated in a given network. The network functions with the greatest number of focus genes that were identified were “Genetic Disorder, Metabolic Disease, Amino Acid Metabolism” and “Lipid Metabolism, Small Molecule Biochemistry, Amino Acid Metabolism” for *E. acervulina* infection, “Lipid Metabolism, Molecular Transport, Small Molecule Biochemistry” for *E. maxima* infection, and “Carbohydrate Metabolism, Lipid Metabolism, Small Molecule Biochemistry” for *E. tenella* infection.

**Table 5:** Network analysis of the transcripts differentially expressed between 1–3 DPI and 4–6 DPI following primary *Eimeria* infection.

*Eimeria*	Associated Network Functions^1^	No. of Focus Genes
*E. acervulina*	Genetic Disorder, Metabolic Disease, Amino Acid Metabolism	26
	Lipid Metabolism, Small Molecule Biochemistry, Amino Acid Metabolism	26
	Lipid Metabolism, Small Molecule Biochemistry, Molecular Transport	23
	Connective Tissue Development and Function, Skeletal and Muscular System Development and Function, Cellular Compromise	19
	Cell Morphology, Cell-mediated Immune Response, Cellular Development	16
*E. maxima*	Lipid Metabolism, Molecular Transport, Small Molecule Biochemistry	22
	Cellular Development, Nervous System Development and Function, Cellular Assembly and Organization	19
	Lipid Metabolism, Small Molecule Biochemistry, Drug Metabolism	15
	Lipid Metabolism, Molecular Transport, Small Molecule Biochemistry	8
	Cellular Growth and Proliferation, Hematological System Development and Function, Inflammatory Response	8
*E. tenella*	Carbohydrate Metabolism, Lipid Metabolism, Small Molecule Biochemistry	27
	Connective Tissue Development and Function, Embryonic Development, Skeletal and Muscular System Development and Function	24
	Developmental Disorder, Neurological Disease, Genetic Disorder	23
	Cell Cycle, Carbohydrate Metabolism, Small Molecule Biochemistry	21
	Cell Cycle, Connective Tissue Development and Function, Cancer	15

1 Datasets were analyzed by Network analysis using IPA software. Networks are listed in descending order of statistical significance with the most significant at the top of each *Eimeria* species grouping.

## Discussion

The monoxenous life cycle of *Eimeria* is complex and involves intracellular, extracellular, asexual, and sexual stages for invasion and reproduction. It is therefore not surprising that avian immune responses against the parasite are correspondingly complicated, involving aspects of innate vs. adaptive, humoral vs. cellular, and passive vs. active immunity [4], [5]. Because the *Eimeria* life cycle is normally completed in 4–6 days, depending on the particular coccidia species, this study was designed to examine variations in gene expression during the first 6 days following primary or secondary infection. Our findings are summarized as follows: (a) whereas infection by all three *Eimeria* species altered the levels of relatively equal numbers of transcripts at 1–3 DPI following primary infection, *E. tenella* elicited the greatest number of altered transcripts at 4–6 DPI post-primary infection, and at all time points following secondary infection, (b) while equivalent numbers of up-regulated transcripts were detected following *E. tenella* primary and secondary infections, relatively fewer mRNA were modulated following primary vs. secondary infection with *E. acervulina* or *E. maxima*, (c) irrespective of the coccidia used for infection, biological pathway analysis identified the altered transcripts as belonging to the categories of “Disease and Disorder” and “Physiological System Development and Function”, and (d) 16 intracellular signal transduction pathways were identified from the differentially expressed transcripts following *Eimeria* infection, several of which are directly relevant to protective immunity, including those for IL-3, IL-4, CD28, nitric oxide, nuclear factor of activated T cells (NFAT), and the B cell receptor.

These results confirm and extend our prior study which identified lymphocyte transcripts that were altered following *E. acervulina* infection of naïve chickens [6]. In addition to modulation of immune-related transcripts, these two reports also suggest that the expression of genes related to cellular metabolism, especially lipid metabolism, are altered during coccidia infection. Correspondingly, following primary *Eimeria* infection, it is the asexual replicative phase that is responsible for the majority of intestinal tissue damage, with negative consequences for nutrient absorption [23]. Therefore, it is not unexpected to detect changes in the expression of genes related to host metabolic function during the primary infection. In particular, several mRNAs encoding proteins with known effects on lipid metabolism were altered by *Eimeria* infection. These include perilipin 2 (PLIN2), which was increased subsequent to primary infection by *E. acervulina* and *E. maxima*, as well as prostaglandin reductase 1 (PTGR1), CD36, and carnitine O-octanoyltransferase (CROT), which were decreased following *E. acervulina* primary infection. Moreover, *E. maxima* infection suppressed the expression of the lipid-related mRNAs for scavenger receptor class B, member 1 (SCARB1), hydroxysteroid (11-β) dehydrogenase 1 (HSD11B1), hydroxysteroid (17-β) dehydrogenase 4 (HSD17B4), solute carrier organic anion transporter family, member 2A1 (SLCO2A1), while *E. tenella* infection decreased that for glycerol-3-phosphate acyltransferase, mitochondrial (GPAM).

Following both primary and secondary infections, *E. tenella* modulated the levels of the greatest number of transcripts compared with uninfected controls (primary, 2,359; secondary, 2,522), as opposed to *E. acervulina* (primary, 195; secondary, 2,431) and *E. maxima* (primary, 213; secondary, 1,717). *E. tenella* is known to cause cecal or “bloody” coccidiosis, and primarily invades the intestinal ceca [24]. Severe intestinal bleeding, eroding of the mucosal surface, and thickening of the cecal wall are all clinical signs of *E. tenella* infection. By 6–8 DPI, rupture of the cecal wall may occur, with associated high mortality [25]. Relevant to this topic, our biological function analysis identified *E. tenella*-elicited transcripts in the category “Hematological System Development and Function” and “Hematopoiesis” of “Physiological System Development and Function”, with 126 and 80 focus genes, respectively. Among these genes related to hematological functions were adenosine deaminase (ADA), BCL2-related protein A1 (BCL2A1), caspase 1, apoptosis-related cysteine peptidase (CASP1), chemokine (C-C motif) receptor 9 (CCR9), chemokine (C-X-C motif) receptor 4 (CXCR4), CD5, CD44, CD69, cyclin D1 (CCND1), IL-6, and IL-10 receptor α (IL10RA).

Whereas *E. tenella* altered the expression of the greatest number of transcripts, compared with uninfected chickens, comparisons between primary and secondary infections revealed the fewest number of modified transcripts for this species. In other words, primary infection with *E. tenella* induced the greatest transcriptional response in intestinal lymphocytes that was maintained during secondary infection. These results imply that primary infection of *E. tenella* may be more likely to induce protective immunity, compared with *E. acervulina* or *E. maxima*. Although the particular immune effector cell(s) involved in protective immunity against individual *Eimeria* species remain to be determined, previous studies showed that depletion of CD4^+^ lymphocytes enhanced primary infection by *E. tenella*, but did not influence the course of *E. acervulina* infection, suggesting that this subpopulation is important in controlling primary infection by the former but not the latter [26]. On the contrary, no differences between the two coccidia were noted following depletion of CD8^+^ cells. Ongoing studies in our laboratory are designed to characterize the transcriptional profiles of CD4^+^, CD8^+^ and other intestinal lymphocyte subpopulations following primary and secondary infection with the different coccidia species.

In summary, this report describes the transcriptional responses of chicken intestinal lymphocytes following *in vivo* experimental infection with *E. acervulina*, *E. maxima*, or *E. tenella* using the AVIELA microarray. Biological function and pathway analysis identified the altered transcripts being relevant to lipid metabolism, as well as cellular and humoral immunity. These new developments further enhance our understanding of the host response to *Eimeria* infection that may someday contribute to the development of the alternative control strategies against avian coccidiosis whose treatment has traditionally relied upon prophylactic medication and antibiotics.

## Supporting Information

Table S1 The annotated transcripts (P<0.0005) that were differentially expressed over the entire 6 day infection periods when comparing each infection group with uninfected controls.
(XLS)

Table S2 The annotated transcripts that were differentially expressed (P<0.01) between early (1–3 DPI) and late (4–6 DPI) primary infections with *E. acervulina*, *E. maxima*, *or E. tenella*.
(XLS)
